# Orthologue chemical space and its influence on target prediction

**DOI:** 10.1093/bioinformatics/btx525

**Published:** 2017-08-26

**Authors:** Lewis H Mervin, Krishna C Bulusu, Leen Kalash, Avid M Afzal, Fredrik Svensson, Mike A Firth, Ian Barrett, Ola Engkvist, Andreas Bender

**Affiliations:** 1 Centre for Molecular Informatics, Department of Chemistry, University of Cambridge, Cambridge, UK; 2 Oncology Innovative Medicines and Early Development, AstraZeneca, Cambridge, UK; 3 Discovery Sciences, AstraZeneca R&D, Cambridge Science Park, Cambridge, UK; 4 Discovery Sciences, AstraZeneca R&D Gothenburg, Mölndal, Sweden

## Abstract

**Motivation:**

*In silico* approaches often fail to utilize bioactivity data available for orthologous targets due to insufficient evidence highlighting the benefit for such an approach. Deeper investigation into orthologue chemical space and its influence toward expanding compound and target coverage is necessary to improve the confidence in this practice.

**Results:**

Here we present analysis of the orthologue chemical space in ChEMBL and PubChem and its impact on target prediction. We highlight the number of conflicting bioactivities between human and orthologues is low and annotations are overall compatible. Chemical space analysis shows orthologues are chemically dissimilar to human with high intra-group similarity, suggesting they could effectively extend the chemical space modelled. Based on these observations, we show the benefit of orthologue inclusion in terms of novel target coverage. We also benchmarked predictive models using a time-series split and also using bioactivities from Chemistry Connect and HTS data available at AstraZeneca, showing that orthologue bioactivity inclusion statistically improved performance.

**Availability and implementation:**

Orthologue-based bioactivity prediction and the compound training set are available at www.github.com/lhm30/PIDGINv2.

**Supplementary information:**

Supplementary data are available at *Bioinformatics* online.

## 1 Introduction

**Fig. 1.**
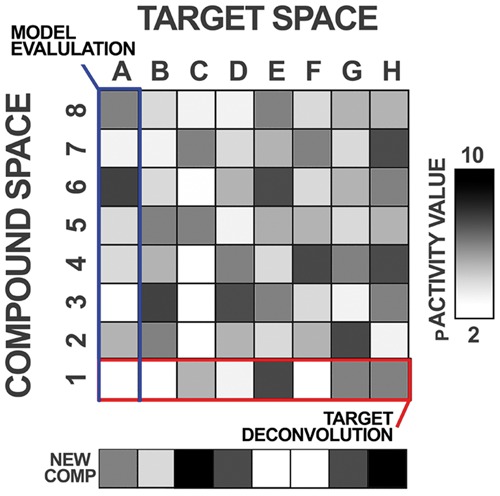
Model evaluation and target deconvolution. Bioactivity data is represented within a matrix of compounds (rows) and targets (columns). Typically, target prediction models are trained and evaluated per target or column-wise (blue), i.e. calculate the performance of a target model given the compounds retrieved. When the models are deployed for target deconvolution (red), the models are interpreted per compound (row), i.e. to identify the targets predicted for a compound. In this work, the evaluation of predictive models is based on the column-wise assessment of predictions for each new compound (shown)

*In silico* deconvolution is a well-established computational technique capable of inferring compound activity using similarity relationships between orphan compounds and identified ligands (Wang *et al.*, 2013). In this technique, target prediction models can be deployed to produce knowledge-based predictions for new, untested ligands across a spread of proteins, as shown in Figure 1. Consequently, it is possible to explore the full bioactivity spectra across all targets available, to identify patterns present in predicted space to generate a mode-of-action hypothesis for a previously unseen molecular structure (Lavecchia and Cerchia, 2016).

Previous approaches often focus within one species, where bioactivity information for a single organism is extracted from bioactivity repositories (Cereto-Massagué *et al.*, 2015; Ivanov *et al.*, 2016). In this situation, annotations for orthologous protein relationships, the closest relative of a given gene in a different species, are disregarded. Since orthologues share functional similarity and are likely to share similar bioactivity profiles, the mapping between species offers an attractive approach to integrate bioactivity between related proteins, to improve the chemical space covered (Dimova *et al.*, 2015). Mapping activity information to other organisms is also valuable since small molecules of therapeutic interest are frequently tested in model organisms before in man.

There are relatively few cheminformatics analyses exploring the impact of incorporating small molecules binding orthologous proteins in a target prediction context (Koutsoukas *et al.*, 2013). One study illustrated the mapping of compound interactions across orthologues improves target prediction accuracy, whilst inclusion of paralogue data was found to worsen the accuracy in some cases (Gfeller and Zoete, 2015). These findings outline the potential advantages of integrating orthologue results across species, but outline limitations for such approaches and the need for deeper investigation.

Various Proteochemometric Modeling (PCM) studies incorporate orthologues to increase the data available for training. One study combined bioactivities from *Rattus norvegicus* with human data points during PCM training, for the identification of novel adenosine receptor ligands (van Westen *et al.*, 2012). Significant amounts of rat data were available due to the historical role of this species in adenosine bioactivity experiments. Protein domain annotations were extrapolated across species for PCM and *in silico* target deconvolution during the retrospective discovery of DHFR inhibitors in *Plasmodium falciparum* (Paricharak *et al.*, 2015). Models performed with recall and precision values of 79 and 100%, respectively. HomoloGene (Coordinators, 2013) has also been employed to improve the coverage of target prediction training data, providing 9 565 534 active and 598 923 798 inactive data points for modelling, spanning 2 882 targets (Mervin *et al.*, 2016).

Other studies conducted analyses into bioactivity space outside of a target prediction context, analyzing compound-ligand annotations between related targets in distinct organisms (Klabunde, 2007). A systematic search for bioactive small molecules shared by orthologous targets identified compound-orthologue pairs, covering 938 orthologues, 358 unique targets and 98 organisms (Dimova *et al.*, 2015). The authors introduced an orthologue compound-target classification system comprising “organism cliffs” and “potency-retaining” pairs. An analysis of ChEMBL data demonstrated a highly significant relationship between bioactivities in human and rat targets (*R* = 0.71, *p *<* *2_e__-__16_), although outliers were identified for targets such as the Histamine H_3_ receptor (Kruger and Overington, 2012). Other work mined *Homo sapiens* bioactivity data together with structural and historical assay space searches, to propose cross-species targets alongside respective hit compounds for the treatment of *M.tuberculosis* (Martínez-Jiménez *et al.*, 2013). One study comprised generation of phylogenetic and bioactivity tree representations of kinases, highlighting clustering targets in protein structure space makes incorrect assumptions about interactions in bioactivity space (Paricharak *et al.*, 2013). In fact, many factors need to be considered when performing homology-based bioactivity inference between kinase targets, illustrating the potential pitfalls of extrapolating ligand interactions between species. Recent advances in the cheminformatics field have also analyzed the influence of other disparate sources of experimental data, showing that even the inclusion of data from random assays can improve the performance of models (Ramsundar *et al.*, 2015).

In this study, we verify the advantages and limitations of leveraging the wealth of orthologue bioactivity data for model generation in a target prediction setting. We address if it is valid to combine orthologue data over the range of targets with available bioactivities in the first instance, by extracting bioactivity data from ChEMBL (Bento *et al.*, 2014) and PubChem (Wang *et al.*, 2009) and explore conflicting annotations between human and orthologue bioactivities. We analyze the concordance of activity values between binding and functional assays in human and orthologue, to test generalizability across bioactivity data. Chemical space analysis is conducted to explore the chemical data added to models. For example, if additional bioactivity data is too similar to existing training data, these compounds are of lower value since no new information is gained. If training data is too dissimilar, singleton bioactivity data points could interfere with the model by diluting the weight of important features.

Finally, we assess the effect of orthologue-based bioactivity inference on model performance and translatability, using a time-series split validation of Random Forest, Naïve Bayes and SVM models, with a range of hyper-parameters in Scikit-learn (Pedregosa *et al.*, 2011), before and after the inclusion of bioactive orthologue data. External validation was performed using compounds from Chemistry Connect (Muresan *et al.*, 2011) and HTS data available at AstraZeneca. The realized models and training set are available at www.github.com/lhm30/PIDGINv2.

## 2 Materials and methods

### 2.1 Compound bioactivity data and pre-processing

Bioactive molecules were extracted from ChEMBL_21 for pChEMBL activity values -Log(K_i_/K_d_/IC_50_/EC_50_) greater than or equal to ‘5’ (10 µm) in binding and functional assays for confidence scores greater than ‘5’ (to ensure protein complexes are included) as cut-off for active bioactivity. Percentage activation and inhibition data was also extracted when the ‘*Activity Comment*’ was declared ‘*active’*. The dataset contains 766 515 active data points, spanning 1 651 human targets.

Orthologue data from HomoloGene was extracted for non-human data points spanning 3 231 targets. Bioactivity data for orthologues were integrated with human bioactivity data with the removal of duplicate SMILES. A total of 114 960 orthologue compounds are added to 927 different models (∼56% of the targets modeled). Targets were filtered for a minimum of 10 training compounds before mapping, to retain proteins encapsulating sufficient chemical space. Novel models are available after orthologue inclusion due to surpassing this threshold, which are discussed in Section 3.

PubChem was mined for inactive compounds in the same procedure to (Mervin *et al.*, 2015) resulting in the extraction of 3 630 485 inactives spanning 1 440 targets. A sphere exclusion algorithm was applied to sample putative inactive compounds for targets with insufficient numbers of inactives (1: 100 active versus inactive ratio) using the protocol in (Mervin *et al.*, 2016). 1 491 615 compounds were sampled in this way for 711 (∼43%) targets, producing an inactive dataset of 5 122 100 molecules.

RDKit (Landrum, 2006) was employed to filter structures without carbon and for atomic numbers between 21 and 32, 36 and 52 and greater than 53, with a molecular weight between 100 and 1 000 Da. Compounds were standardized with ChemAxon Standardizer (ChemAxon, 2015), with options set to ‘*Remove Fragment*’ (keep largest), ‘*Neutralize*’, ‘*RemoveExplicitH*’, ‘*Clean2d*’, ‘*Mesomerize*’ and ‘*Tautomerize*’. ChEMBL target-compound pairs were retained when conflicting inactive bioactivities arose, since this data is manually curated.

### 2.2 Target prediction methodology

2 048-bit ECFP_4 binary Morgan fingerprints, radius of 2 atoms, were generated in RDkit for pre-processed molecules. Scikit-learn Random Forest (RF) classifiers of ‘*5*, ‘*50*’ and ‘*500*’ trees, with ‘*n_ features*’ and *‘max_depth’* set to ‘*auto*’ and ‘*class_weight*’ set to ‘*balanced*’, were trained on the fingerprints of active and inactive compounds on a per target basis, whilst supplying the ‘*fit’* method the *‘sample_weight’* of the active versus inactive compound ratio. Bernoulli Naïve Bayes (BNB) models were trained in Scikit-learn with ‘*alpha*’ values of ‘*1.0*’ and ‘*0.1*’. Lineaer SVM classification models (SVC) were also trained using Scikit-learn with the kernel set to ‘*linear*’ and ‘*C*’ penalty parameters of ‘*1.00e-02*’, ‘*1.00e+0*’, ‘*1.00e+2*’. Platt scaling was performed using the Scikit-learn class ‘*CalibratedClassifierCV*’ with n_folds set to ‘*2*’ whilst supplying the ‘*sigmoid*’ method parameter (Platt, 1999). Platt scaling ensures the outputs of target models are calibrated to reflect a degree of confidence of the predicted label. This aims to address the applicability domain, since probabilities from well calibrated classifiers can be interpreted at a confidence level with predictions specified at an acceptable error rate (Pedregosa *et al.*, 2011).

### 2.3 Time series split and external performance evaluation

The Scikit-learn class ‘*TimeSeriesSplit*’ with ‘*n_splits’* set to ‘*5*’ was used to perform five-fold time series split cross validation (CV). Chemistry Connect (Muresan *et al.*, 2011) at AstraZeneca was employed as an external source of bioactivity testing data for activity values (K_i_/K_d_/IC_50_/EC_50_) less than or equal to 10 μM. Duplicate bioactivities to training data were removed, giving 3 061 461 active compounds, covering 572 proteins. 183 099 194 inactive compound-target pairs (distinct from PubChem) were extracted from 420 AstraZeneca target-based screens, spanning 88 GPCRs, 77 kinases and 31 proteases. Sphere exclusion sampled 3 479 469 putative inactive compounds at AstraZeneca, for targets with insufficient in-house inactivity data. PR-AUC, BEDROC (‘*alpha’* = ‘*20*’) (Truchon and Bayly, 2007) and precision, recall and F_1_-Scores (at a *p*(*activity)* greater than 0.5) were calculated across all folds per target during internal or external validation.

## 3 Results

### 3.1 Exploratory analysis of orthologue bioactivity data

The distribution of orthologue compounds incorporated into models separated by organism and target class is shown in Supplementary Table S1, indicating the frequency and distribution of bioactivities differ between organism and target class. *Rattus norvegicus* (rat) and *Mus musculus* (mouse) contribute the largest number of compounds among the orthologues with 77 156 and 29 119 data points (∼67 and ∼25%), respectively. Although popular for *in vivo* studies, *Canis lupus familiaris* (dog) comprises fewer orthologue target-compound pairs (433), due to the smaller number of biochemical assays conducted *in vitro* for this species. Rat, mouse and *Bos taurus* (bovine) contribute 30 814, 3 485 and 1 563 compounds to GPCRs (across 185 targets), making it the largest target class in terms of orthologue compounds included. Ion channels are ranked second, comprising 22 223 bioactive compounds from orthologues, although they represent a class where the bulk of mapped data points (20 713 compounds for 116 targets) are derived from rat. The Lyase target class is dominated by bovine data, which contributes 1 179 of the 1 314 bioactivities from orthologues. 1 101 of these are annotated for ‘Carbonic anhydrase 4’ (CA4), which originate from the popular purification method of CA4 extraction from bovine lung tissue (Scozzafava *et al*., 2012). NHRs, ranked third, are dominated by ‘Nuclear receptor ROR-gamma receptors’ (RORC), due to a human-orthologue pair between ‘P51449’ and ‘P51450’, with data from two PubChem qHTS assays (CHEMBL1614441 and CHEMBL1614087) comprising 11 781 and 7 452 actives, respectively. For contrast, a ROR training set comprising only human data would only include 418 active compounds.

Although CA4 and RORC dominate their respective target classifications with ∼84 and ∼96% of orthologue compounds, this should not de-emphasize the impact of minor amounts of bioactivity data incorporated into under-represented targets. Indeed, one important aspect to consider when generating *in silico* target predictions are biases towards certain protein classifications due to the irregular distribution of data points in *validated* bioactivity space, which are projected forward into *predicted* bioactivity space. For example, GPCRs and kinases are examples of well-studied target classifications where the abundance of data leads to their significant representation in the targets modeled. One case for orthologue inclusion is the potential to relax such biases in predicted bioactivity space by increasing the numbers of targets surpassing the minimum threshold for training data. Supplementary Table S2 shows there are 51 target models which would otherwise contain too few actives without bioactivity data from orthologues. The inclusion of these models alleviates the previous biases of target classes to some extent *via* improving the representation of minority classes, with better representation for hydrolases (+9.17%), transporters (+8.05%) and ion channels (+7.21%). There are lower (or no) increases for many previously dominating classes, such as kinases (+0.00%), proteases (+2.14%) and GPCRs (+2.49%).

Another application of target prediction is the extension of predicted bioactivity profiles with gene-pathway and gene-disease associations, to better rationalize the mode-of-action of compounds (Mohamad Zobir *et al.*, 2016). On this topic, we analyzed the impact of the new 51 models, in terms of the improved pathway coverage from BioSystems (Coordinators, 2013) and diseases from DisGeNET (Piñero *et al.*, 2017). Results show 62 newly accessible pathways are available when deploying models, with enhanced coverage for 1 861 pathways. There are 95 novel diseases with improved coverage for 1 388 annotations (Supplementary Table S3). Many diseases are well-studied, such as ‘Breast Carcinoma’, ‘Schizophrenia’ and ‘Colorectal Cancer’, highlighting the real-world significance for using orthologue bioactivity space to expand coverage.

This section highlights the potential benefits for including bioactive orthologues in terms of the proportion of data points available for modelling and the effect this has on the number of targets, pathways and diseases encapsulated by the realized models.

### 3.2 Chemical space of the orthologue data

**Fig. 2.**
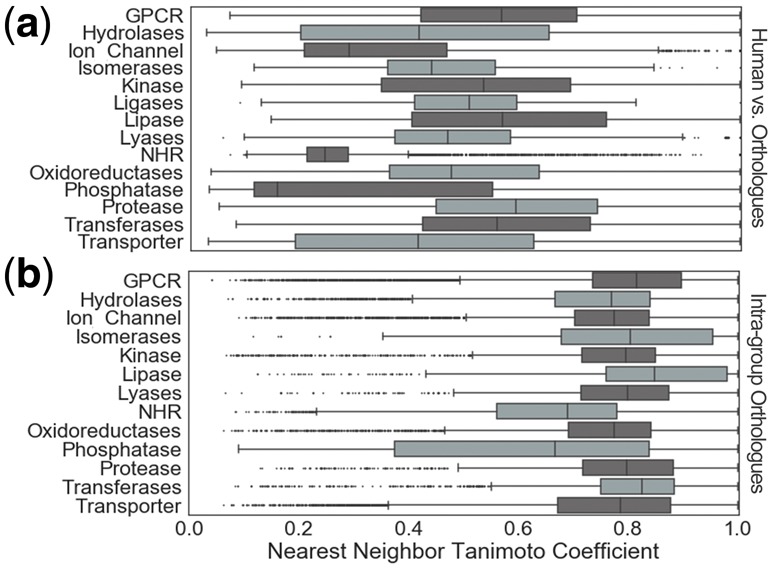
Nearest neighbor analysis. Tanimoto coefficient of ECFP_4 fingerprints were used to define similarity. (**a**) Human and orthologue nearest neighbor similarity indicates the chemical spaces covered by both datasets are dissimilar. This suggests that orthologue data could effectively extend the chemical space of models. (**b**) Nearest neighbor analysis indicates orthologues are frequently similar to each other. High intra-group similarity is suited to modelling, since this enables models to better identify key features

We next analyzed the ECFP_4 Tanimoto coefficient (Tc) nearest neighbor (NN) similarity between active compounds in orthologue and human, since the intention of the orthologue mapping is to also extend the chemical space modeled. Results of the analysis are shown in Figure 2a, indicating the inclusion of bioactive orthologues improves the diversity of training data by incorporating dissimilar areas of chemical space. Almost half (49.6%) of the compounds have a Tc NN similarity to human compounds less than 0.4. This is a surprising degree of dissimilarity, considering one study showed 95% of the actives from ChEMBL have a NN Tc similarity greater than 0.424 for human targets (Mervin *et al.*, 2015). Results indicate similarity differs by target classification where the disparate chemistry for NHRs, a median Tc of 0.22, can be contrasted with the relatively similar bioactive compounds tested for proteases with a Tc of 0.59. The dissimilar chemistry tested between species may arise from the varying organism specific ADMET properties dependent on the specific use of compounds at a given target. We also examined the similarity of compounds within the groups of orthologues available for each target, to elucidate intra-group chemical diversity. Figure 2a shows orthologues are more similar to each other than when compared to their human neighbor counterparts in Figure 2b, indicating they may be useful for training since they do not comprise many singleton compounds.

Here we have shown the chemical space contributed by orthologue data is diverse and novel to existing human training data. We hence expect that integration of both datasets will add value to the compound training set to be modeled.

### 3.3 Conflict analysis of orthologue data

One important question when employing orthologue-based bioactivity inference is the accuracy of assuming shared bioactivity between orthologues and human targets. To address this question, we explored the number of cases when a compound is active in ChEMBL but represented as inactive in PubChem screens in orthologue and *vice-versa*. The two sets of results from the comparison between conflicting orthologue active and inactive bioactivities are shown in Supplementary Table S4.

In total, 1 363 of the 124 540 orthologue bioactivities (spanning 206 HomoloGene protein mappings) have conflicting annotation with the respective compounds inactive in human, indicating conflicting bioactivities in 1.2% of cases according to the analysis presented here. At the target-mapping level, 650 of the 856 human-orthologue HomoloGene target pairs (75.9%) do not comprise conflicting annotations with inactive compounds.

The top 10 conflicting orthologue active and inactive human bioactivities are shown on the left of Supplementary Table S4, indicating the top targets comprise GPCRs (4), NHRs (2) and Ion Channels (2). The proportion of conflicting bioactivities vary widely between the top-ranking targets, and absolute numbers of disagreements are sensitive to the size of existing human data. For example, ‘P35372’ to ‘P33535’, ranked first, comprises 85 conflicting bioactivities, which is proportionately low compared to the 2 291 orthologues mapped (∼4% conflict). In comparison, ‘Q00613’ to ‘P38532’, ranked sixth, comprises 38 conflicting bioactivities and a larger proportion of the 46 orthologue actives mapped (∼83% conflict).

Multiple sequence alignment was conducted using CLUSTAL Omega (Version 1.2.4) for the most frequently conflicted targets, since this is one possible reason for differential activity. The ‘CLUSTAL Sequence Similarity %’ column in Supplementary Table S4 comprises results from this analysis, indicating high numbers of conflicting bioactivities are not necessarily correlated with low sequence similarity. This observation is likely influenced by the nature of the alignment, since the active site is unannotated for many top ranking Uniprot accessions and alignments are forced to reflect amino acid changes outside ligand binding domains. Our results are further affected by the requirement for compounds to be tested in both organisms *and* for these measurements to show differential activity, which is subject to separate testing biases.

The most conflicted target between the human actives and orthologue inactives is the GPCR, ‘Mu-type opioid receptor’ (OPRM1), comprising HomoloGene mapping between human ‘P35372’ and rat ‘P33535’. Alignment identifies these two targets as 93.75% similar, with 7 substitutions within the 400 and 398 amino acid sequences in the binding domain of both targets. Although these changes may be responsible for the differential activity and conflicting bioactivity data, this stipulation requires follow-up testing to corroborate, and the reason for conflicts may also result from various other factors. One example is annotation error, since chemogenomic repositories are known to be plagued by both supplier-specific and repository-specific annotation error rates (Tiikkainen *et al.*, 2013). Additionally, inactivity mining uses the ‘activity_outcome’ bioactivity flag in PubChem, which has been shown to comprise conflicting bioactivities with ChEMBL actives within the same organism (Mervin *et al.*, 2015).

Androgen Receptor (AR) ‘P10275’ to ‘P15207’ is the only orthologue-target pair within Supplementary Table S4 comprising X-ray crystal structures for both proteins, affording an opportunity to superimpose structures between human and orthologue binding sites to visualize changes responsible for differential activity (Supplementary Fig. S1). We ascertain the sequences of residues 670–920 within the ligand binding domain are identical in both organisms, with high structural overlap. Thus, we stipulate AR conflicts are due either to flexibility in more distant regions of the protein, a set of allosteric binding compounds with affinity at the protein domain not part of the crystal structure (full alignment similarity of 84.44%), or misannotation of compound activity.

In a complementary analysis, we validated the reversed mapping of orthologue activities, where inactive orthologues from PubChem are compared to activities from ChEMBL. Overall, 860 of the 3 629 661 inactive compound mappings overlap with actives from ChEMBL, for 134 of the 420 orthologue target pairs. The top 10 targets from this analysis are shown on the right of Supplementary Table S4. Nuclear receptor coactivator 3 (NCOA-3) ‘Q9Y6Q9’ is ranked first, where 138 of the 290 700 mapped inactives have conflicting bioactivities with actives (∼75.4% overlap).

**Fig. 3.**
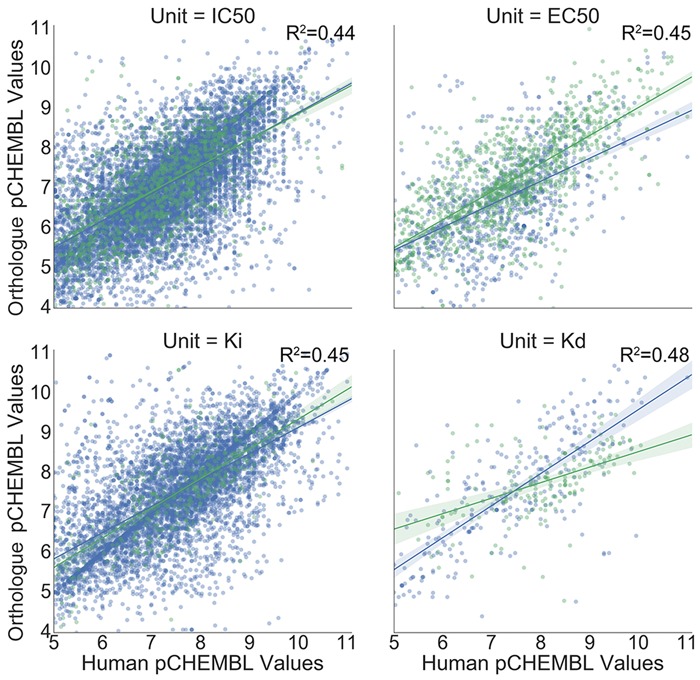
Correlation of human and orthologue pChEMBL values. Bioactivity correlation varies between units of measurement and the type of assay. 21 446 compounds are tested in human and orthologue binding (blue) and functional assays (green). The linear regression line is shown with a 95% confidence interval. *R*^2^ reflects correlation per *Unit*

To test how often differential affinity of ligands between orthologues exists in experimentally confirmed results and the validity of the orthologue-based bioactivity hypothesis in general, we analyzed the concordance between human and orthologue pChEMBL values grouped by the ‘*standard_units*’, ‘*standard_type*’ and ‘*assay_type*’ ChEMBL fields. The results, shown in Figure 3, indicate an overall *R*^2^ of 0.455 between bioactivities observed in human and in orthologues. Overall there is a median pChEMBL discordance of 0.51 between human and orthologue, which is comparable with the median discordance of 0.48 observed between laboratory measurements for proteins within the same organism, and 0.42 after discriminating between assay type (analysis shown in Supplementary Fig. S2). Overall, 20 608 of the 21 446 compounds (96%) would be considered active in both human and orthologue using an activity cut-off of greater than five. A global pChEMBL concordance analysis separated by orthologue species, shown in Supplementary Figure S3, also highlighted that highly concordant affinities in species such as *Gallus* and *Macaca mulatta* can be contrasted with more divergent affinities such as *Arabidopsis thaliana*, suggesting which organisms should be prioritized into target prediction models in the future.

Eight of the ten most discordant pChEMBL values, shown in Supplementary Table S5, exhibit different ChEMBL confidence scores between the compared experiments, indicating discordant measurements may be exaggerated due to comparisons between affinity values obtained at protein complexes and isolated proteins. A docking case study analysis (provided in Supplementary Fig. S4) was conducted for the Nitric oxide synthase orthologue-target pair ranked third, since ‘P29475’ and ‘P29476’ comprise X-ray crystal PDB structures and both activities are annotated for the isolated protein (ChEMBL confidence scores ‘*8*’ or ‘*9*’). CHEMBL526688 is predicted to bind to different amino acid residues in the human and rat binding sites, thus we stipulate differential activity is due to different observable amino acid changes and binding dynamics within the orthologous protein.

On this topic, we sought to analyze the influence of the ‘*Stats-prot-change*’ field in the HomoloGene repository, measuring the ratio of amino acid differences (which is only one possible reason for differences in bioactivity), and the magnitude of disagreement between human and orthologue pChEMBL values. Supplementary Figure S5 shows a binned box plot of ‘*Stats-prot-change’* between the human and orthologue targets and the magnitude of difference between pChEMBL values. We find no trend (*R*^2^ of 0.047) between the magnitude of pChEMBL discordance and the ratio of amino acid differences, although there is an increase in median pChEMBL discordance from 0.490 to 0.550, 0.705 and 1.19 within the ‘*Stats-prot-change’* bins of 0.1, 0.2, 0.3 and 0.4, respectively. One important aspect to consider when interpreting this analysis is that the ‘*Stats-prot-change’* field does not specifically consider the active site of targets, and a signal responsible for divergence or overlap between orthologues may not be encapsulated in this metric.

This section indicates the frequencies of bioactivity conflicts are comparatively low between human and orthologue, and that annotations are overall compatible. We suggest conflicts may also originate from the method of extraction and annotation error, in addition to differential affinity between proteins. We also indicate the choice of species and the ‘*Stats-prot-change*’ metric could help prioritize higher confidence bioactive orthologues in the future.

### 3.4 Time series cross validation split of the models

**Fig. 4.**
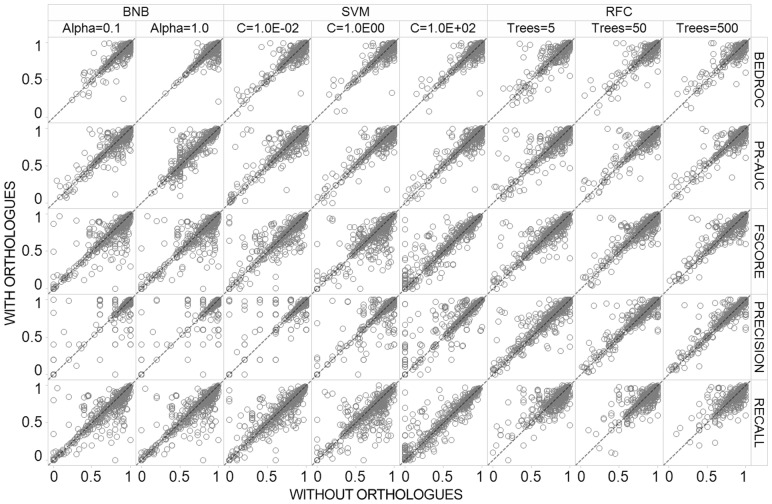
Five-fold time split cross validation (CV) performance. The influence of orthologue inclusion varies between algorithms and hyper-parameter settings

The CV performance of the RF, BNB and SVC algorithms before and after the inclusion of orthologue training data are shown in scatter plot Figure 4, distribution shown in Supplementary Figure S6 and in tabular form in Supplementary Table S6. Overall, the results show that the influence of orthologue inclusion differs both between and within algorithms due to different hyper-parameter settings. For example, the SVM (C = 1.0E+02) showed the largest improvement upon orthologue inclusion, with ∼64% of the models showing stable or improved predictions with median F_1_-Score increased from 0.61 ± 0.35 to 0.67 ± 0.34. Conversely, only ∼40% of the SVM (C = 1.0) target prediction models showed improved or stable performance, with a decreased median F_1_-Score from 0.84 ± 0.25 to 0.81 ± 0.25 (which is particularly influenced by decreased median recall from 0.78 ± 0.26 to 0.72 ± 0.25), highlighting the extent that the hyper-parameter selection influences the benefit of orthologue inclusion and model performance even within the same machine learning algorithm. In comparison, the RFC showed steady performance after addition of orthologues across all tested hyper-parameters, where F_1_-Score performed within two decimal places across all 5, 50 and 500 tree hyper-parameter settings. Thus, the RFC has the highest capacity for maintaining the highest performance across multiple hyper-parameters despite increasing the number of data points comprising diverse chemistry from disparate species.

The distribution of BEDROC performance shows a different behavior to the one obtained for F_1_-Score, since this metric is affected by orthologue bioactivity to a lesser extent. For example, all eight of the tested algorithms and hyper-parameter settings demonstrated similar performance with and without bioactive orthologues within two percent (three of the eight are identical). This reveals that orthologue inclusion mainly improves models by increasing precision and recall (and hence F_1_-Score), but influences the early recognition of the models to a minor degree. This finding is likely influenced by the Platt scaling procedure which aims to better calibrate the probability output by the algorithms, thus models may produce well-adjusted probabilities with good early recognition (BEDROC) performance both before and after orthologue inclusion.

Supplementary Figure S7 illustrates internal CV model F_1_-Score split by target classification, which enabled us to examine the NHR target class, since chemical space analysis previously highlighted that bioactive compounds in orthologue NHRs are both dissimilar from compounds tested in human and diverse from one another. Our analysis outlines this protein classification comprises the largest decrease in F_1_-Score between all algorithms, with only 25% of models showing increased or stable performance across all hyper-parameters (40 out of 160 models tested). Additionally, we have outlined the number of conflicting bioactivities for the NHR class is low, with low discordance of pChEMBL values. Based on this knowledge, we stipulate the observed CV decrease for NHRs is likely due to the limited number of novel structures with dissimilar chemistry from actives at each sampling round (rather than the addition of noise from erroneous data points), thus the implemented algorithms are unable to efficiently distinguish orthologue actives from human inactive training points.

Although some target models are negatively affected by orthologues, this is not automatically a concern for the external application of the models since the compounds responsible for performance decrease will be incorporated into complete training sets, to potentially improve the chemical space of deployed models. We will dissect the performance of these models when extrapolating predictions to the novel chemistry in the AstraZeneca bioactivity data, to assess whether orthologues add value to realized models or form singleton data points confounding the models.

Overall, we show that orthologues incorporate novel chemical diversity into the training set without a significant negative impact to performance overall. Results support the view that compounds active in orthologous targets could enable future predictions to capitalize on extended chemical space, to produce superior predictions with better extrapolation to novel chemistry.

### 3.5 External validation using AstraZeneca bioactivity data

**Fig. 5.**
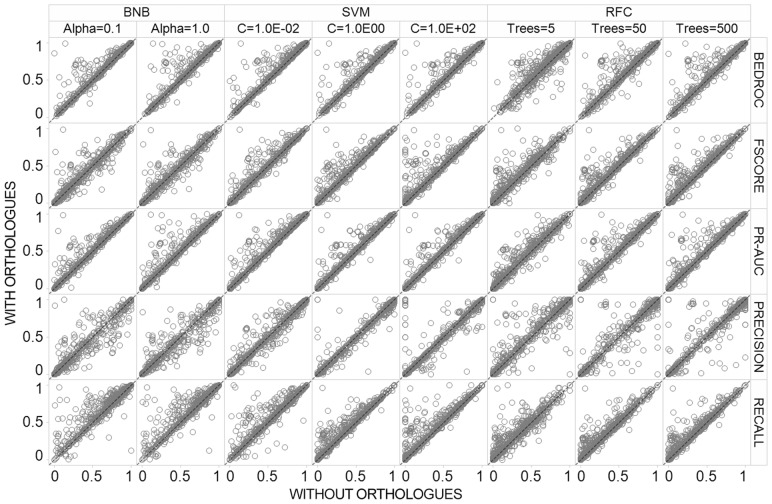
AstraZeneca external validation performance. Decreased performance towards the bottom left of the plots arises from difficult classification instances in external testing compounds, for compounds that are distinct from the training set. In accordance with internal CV, the influence of orthologue inclusion on performance varies between algorithms and hyper-parameter settings

We analyzed the performance of the models for AstraZeneca compounds unrepresented in the training data using bioactive compounds from Chemistry Connect and inactive compounds from HTS screens. The performance from this analysis is shown in the scatter plot Figure 5, distributions in Supplementary Figure S8 and in tabular form in Table 1 and Supplementary Table S7. Our findings highlight decreased performance both before and after orthologue inclusion compared to CV results (markers towards the *bottom left* of the plots), which arises from difficult classification instances when external testing compounds are distinct from the training set.

**Table 1.** Averaged F_1_-Score results for AstraZeneca external validation

Learner	Hyper-parameter	Without orthologues	With orthologues
BNB	Alpha = 0.1	0.33 ± 0.29	0.34 ± 0.29
	Alpha = 1.0	0.35 ± 0.29	0.36 ± 0.29
SVM	C = 1.0E-02	0.44 ± 0.29	0.46 ± 0.30
	C = 1.0E-00	0.38 ± 0.28	0.42 ± 0.28
	C = 1.0E+02	0.39 ± 0.27	0.42 ± 0.27
RFC	Trees = 5	0.33 ± 0.27	0.36 ± 0.27
	Trees = 50	0.37 ± 0.28	0.40 ± 0.28
	Trees = 500	0.37 ± 0.28	0.41 ± 0.28

Compared to CV, the overall distribution of median F_1_-Score external validation performance shows stable or improved scores across all eight of the benchmarked algorithms and hyper-parameters after incorporation of orthologue bioactivities. In corroboration with CV findings, the scatter plot highlights that the choice of algorithm and hyper-parameter influences the effect of orthologue inclusion on model performance to different extents. The SVM (C = 1.0E+02), which was the highest improved algorithm and hyper-parameter for the CV findings, also comprises the largest F_1_-Score increase from 0.35 ± 0.28 to 0.43 ± 0.28 after including orthologue bioactivity (which is due to increased recall scores from 0.42 ± 0.29 to 0.47 ± 0.27). The RFC (Trees = 500) comprises the second largest improvement in F_1_-Score performance from 0.35 ± 0.28 to 0.41 ± 0.28, which is also the highest performing class during external validation.

The performances of the benchmarked models split by target classification are visualized in Supplementary Figure S9. In comparison to CV, many of the markers lie above the diagonal line, indicating benefit of orthologue bioactivity space. Ion channels are the most improved class overall, with large increases across all eight of the algorithms and hyper-parameters benchmarked, with the largest increase obtained by the SVM (C = 1.0E+02) for F_1_-Scores from 0.18 ± 0.19 to 0.35 ± 0.22. We next explored the NHR class, which was previously outlined as problematic across benchmarked algorithms during CV due to the dissimilar orthologue chemistry that was not successfully combined into models during time series split validation. In comparison to CV, the external performance of the NHR is improved for ∼53% of the models (more than twice the number obtained during CV). The largest increase for this class was observed for the BNB (Alpha = 0.1) models with a median F_1_-Score increase of 0.18 ± 0.19 to 0.35 ± 0.22. This result indicates many of the previously problematic orthologue compounds have been successfully incorporated into the realized models to produce superior performance during AstraZeneca bioactivity external dataset validation.

Although the overall distribution and median scores obtained from this analysis show general improvement across the breadth of models, there are many models which are negatively affected by orthologues across the algorithms and hyper-parameters tested, which emphasizes the need to individually assess orthologue space when incorporating this type of bioactivity data points into models. One reason for decreased performance may be due to the uneven distribution of orthologues in chemical space. The addition of singleton compound fingerprints may interfere with the machine learning algorithm by diluting important features as it attempts to accommodate islands of activity from orthologue space. Additionally, larger numbers of orthologues can effectively convert a human target model into an orthologue model containing fewer human bioactivities than ones originating from related organisms.

Our results show that combination of orthologue data across all models should not be performed. Instead, the per-target performance upon orthologue inclusion for each algorithm and hyper-parameter should be assessed, to ensure an informed decision is taken regarding the addition of bioactive compounds from orthologue species into the realized predictors.

## 4 Discussion

Here we present an in-depth analysis of orthologue bioactivity data and its relevance and applicability towards expanding compound and target bioactivity space for predictive studies. We compared the number of conflicting bioactivities between human actives and orthologue inactives and *vice-versa*, indicating annotations are compatible in 98.90 and 99.97% of cases. pChEMBL concordance analysis outlines bioactivity agreement varies between species and indicates which organisms could be prioritized to supplement future models. The HomoloGene ‘prot-stat-change’ could be used to remove discordant target mappings and could also help direct the prioritization of future bioactivity assays, since higher confidence annotations with concordance may not require subsequent profiling in human and *vice-versa*. Chemical space analysis indicated the chemistry contributed by orthologue data is both diverse and novel to existing human training data, suggesting integration of both datasets could add value to the training set to be modeled.

We explored the impact of orthologous bioactivity information on target prediction models using the Random Forest (RF), Bernoulli Naïve Bayes (BNB) and Support Vector Machine Classifier (SVC) algorithms using five-fold time series cross validation (CV). Overall, results showed bioactive orthologues incorporate novel and diverse chemistry into the training set without impacting CV performance, supporting the view that orthologues enable future predictions to capitalize on extended chemical space. External AstraZeneca bioactivity data showed orthologue inclusion significantly increased performance across all machine learning algorithms and hyper-parameters, by enabling the realized predictors to access new chemical space. We illustrate the influence of orthologues on predictivity varies between organism and protein classification due to the quantity and diversity of human and orthologue bioactivities. Ideally, the decision whether to add orthologues could be considered on a per target basis, considering the chemical diversity of human and orthologue data and the biology or disease of interest.

## Supplementary Material

Supplementary Figure S1

Supplementary Figure S2

Supplementary Figure S3

Supplementary Figure S4

Supplementary Figure S5

Supplementary Figure S6

Supplementary Figure S7

Supplementary Figure S8

Supplementary Figure S9

Supplementary Table S1

Supplementary Table S2

Supplementary Table S3

Supplementary Table S4

Supplementary Table S5

Supplementary Table S6-S7

Supplementary Figure Legends
