# Supplementary figures and images for: Orthologue chemical space and its influence on target prediction
Source: Bioinformatics. 2017 Aug 26;34(1):72–9. doi: 10.1093/bioinformatics/btx525 (PMC5870859; doi:10.1093/bioinformatics/btx525)

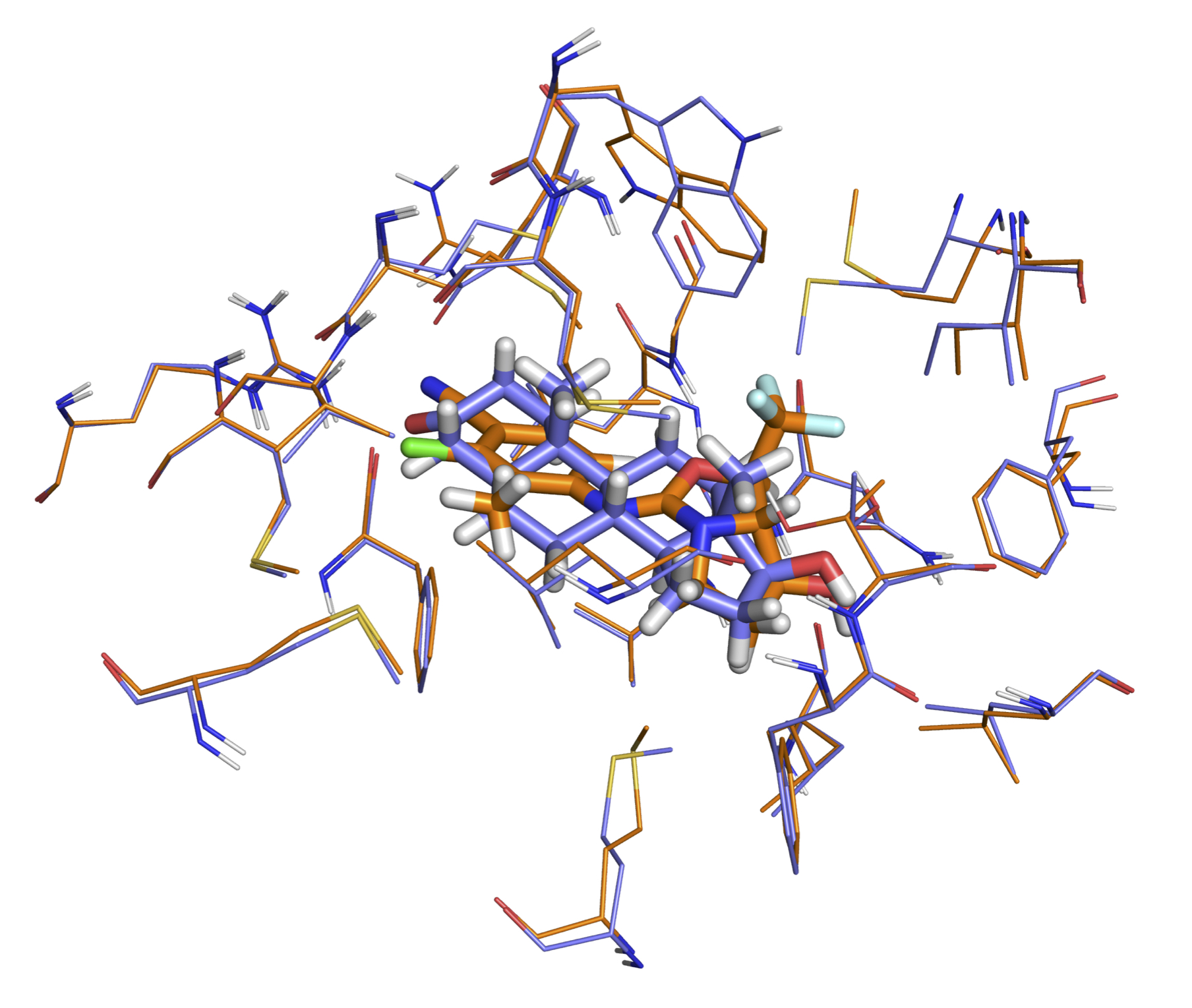

Supplement: Supplementary Figure S1 [file sf1_btx525.jpeg]

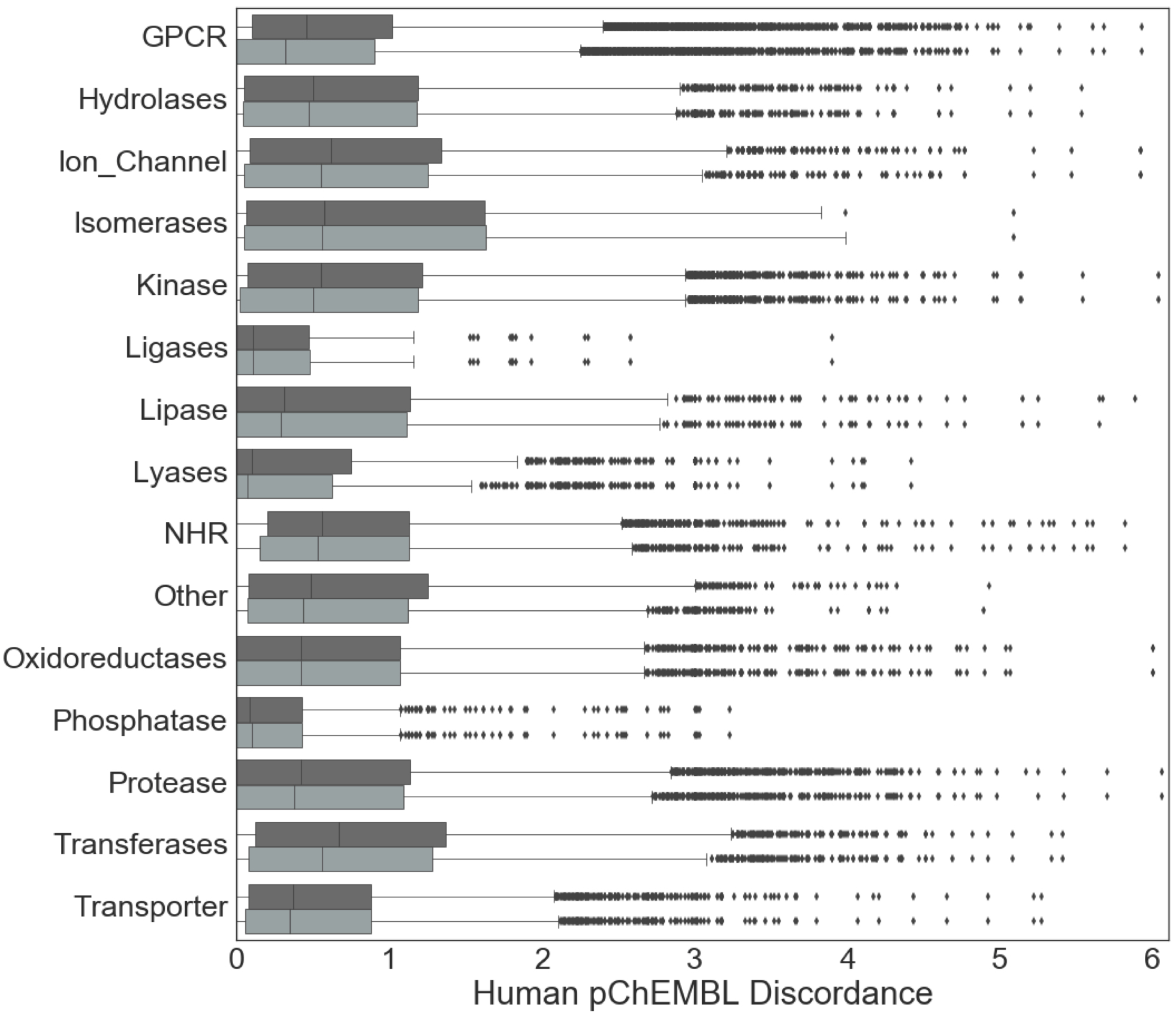

Supplement: Supplementary Figure S2 [file sf2_btx525.jpeg]

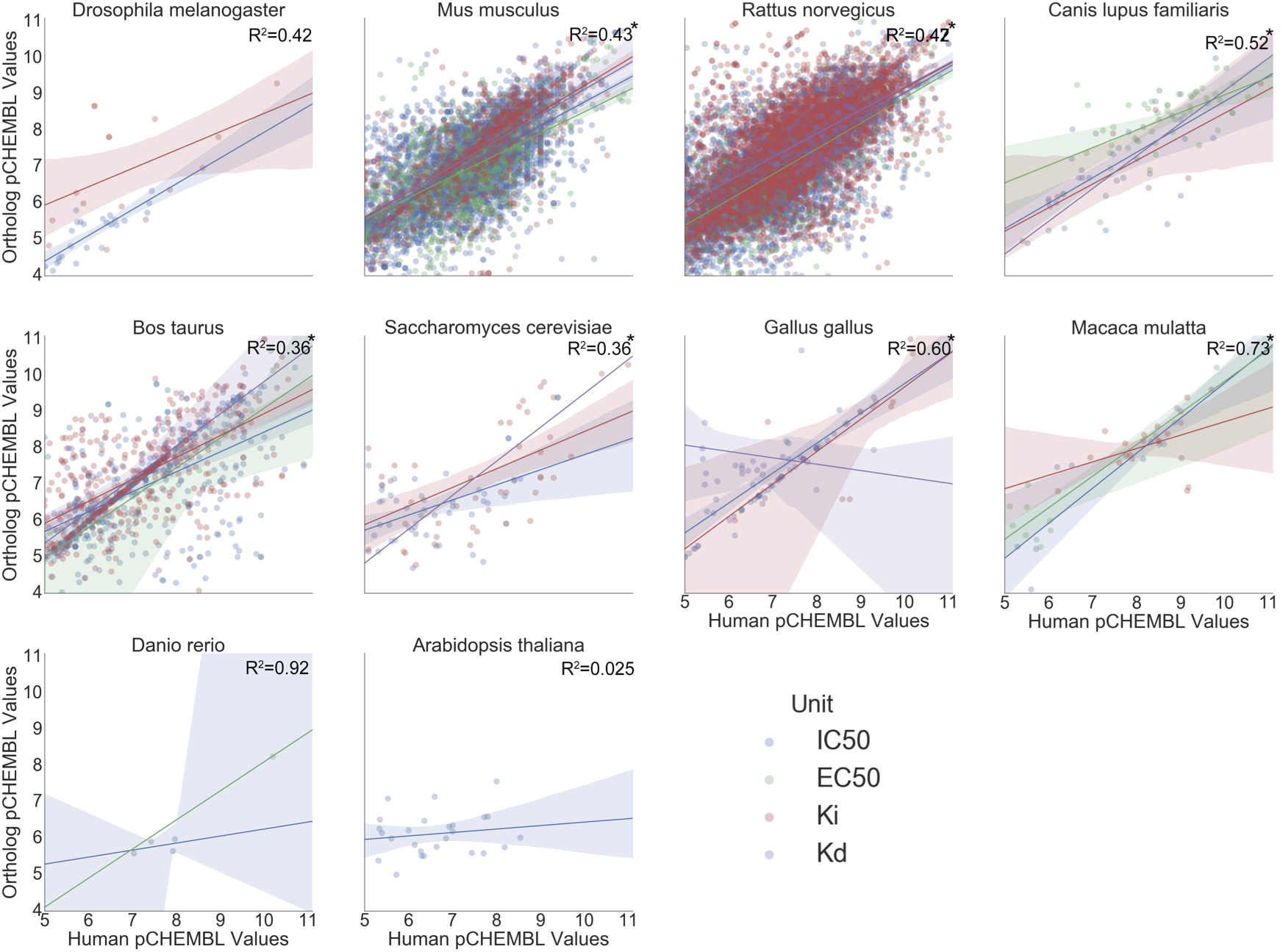

Supplement: Supplementary Figure S3 [file sf3_btx525.jpeg]

a)

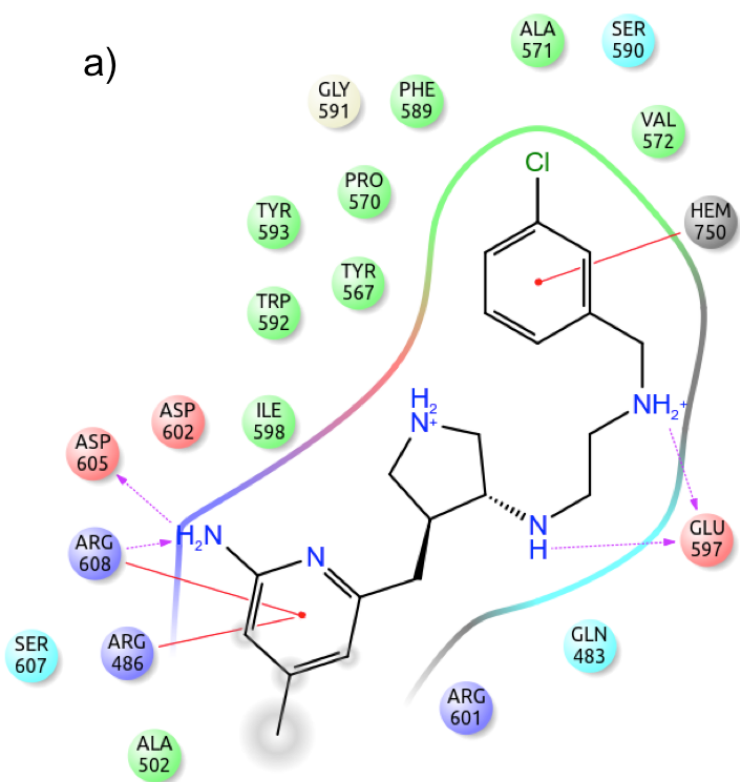

b)

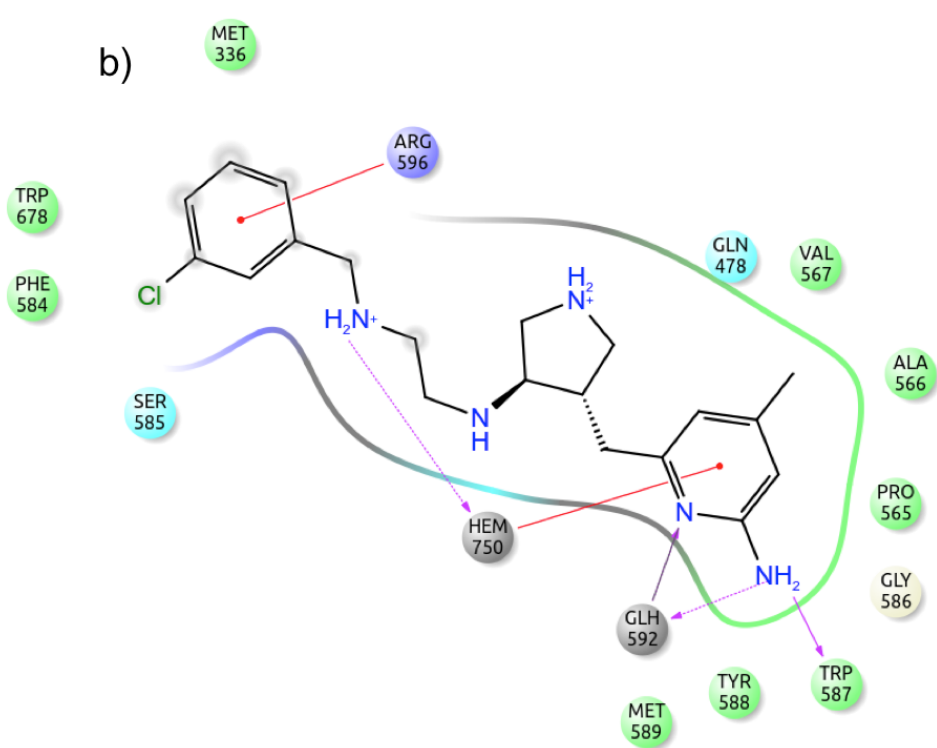

Supplement: Supplementary Figure S4 [file sf4_btx525.pdf]

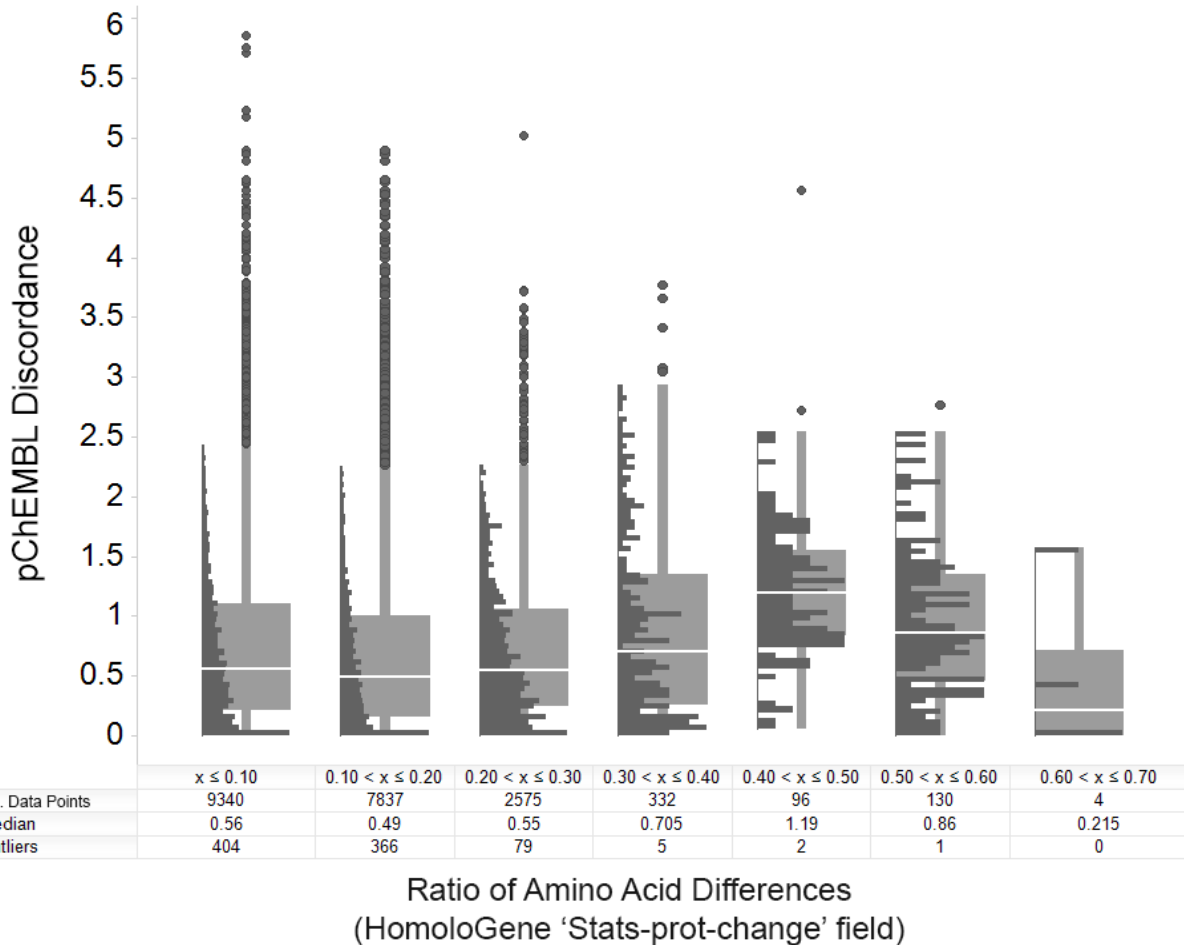

Supplement: Supplementary Figure S5 [file sf5_btx525.pdf]

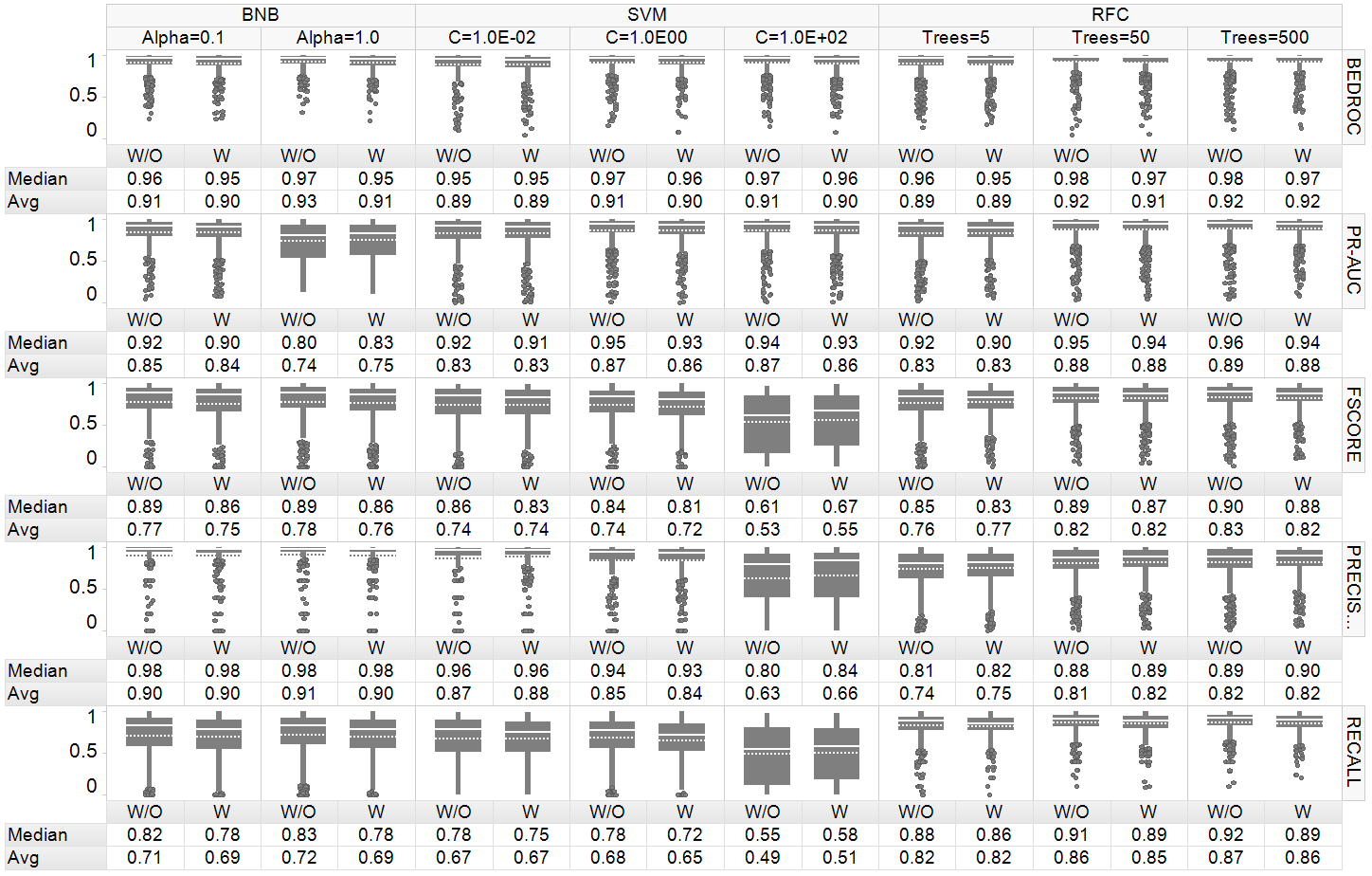

Supplement: Supplementary Figure S6 [file sf6_btx525.png]

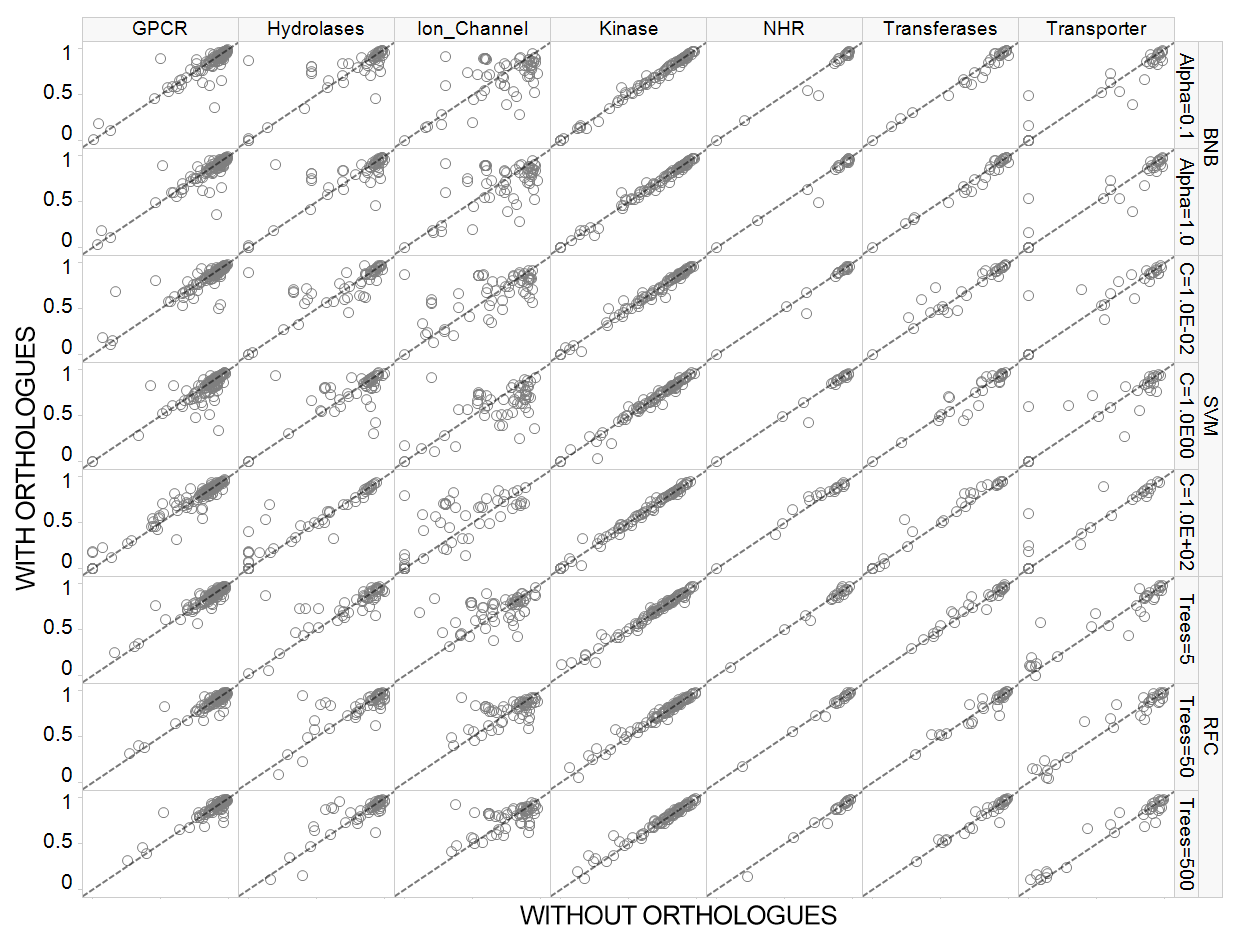

Supplement: Supplementary Figure S7 [file sf7_btx525.png]

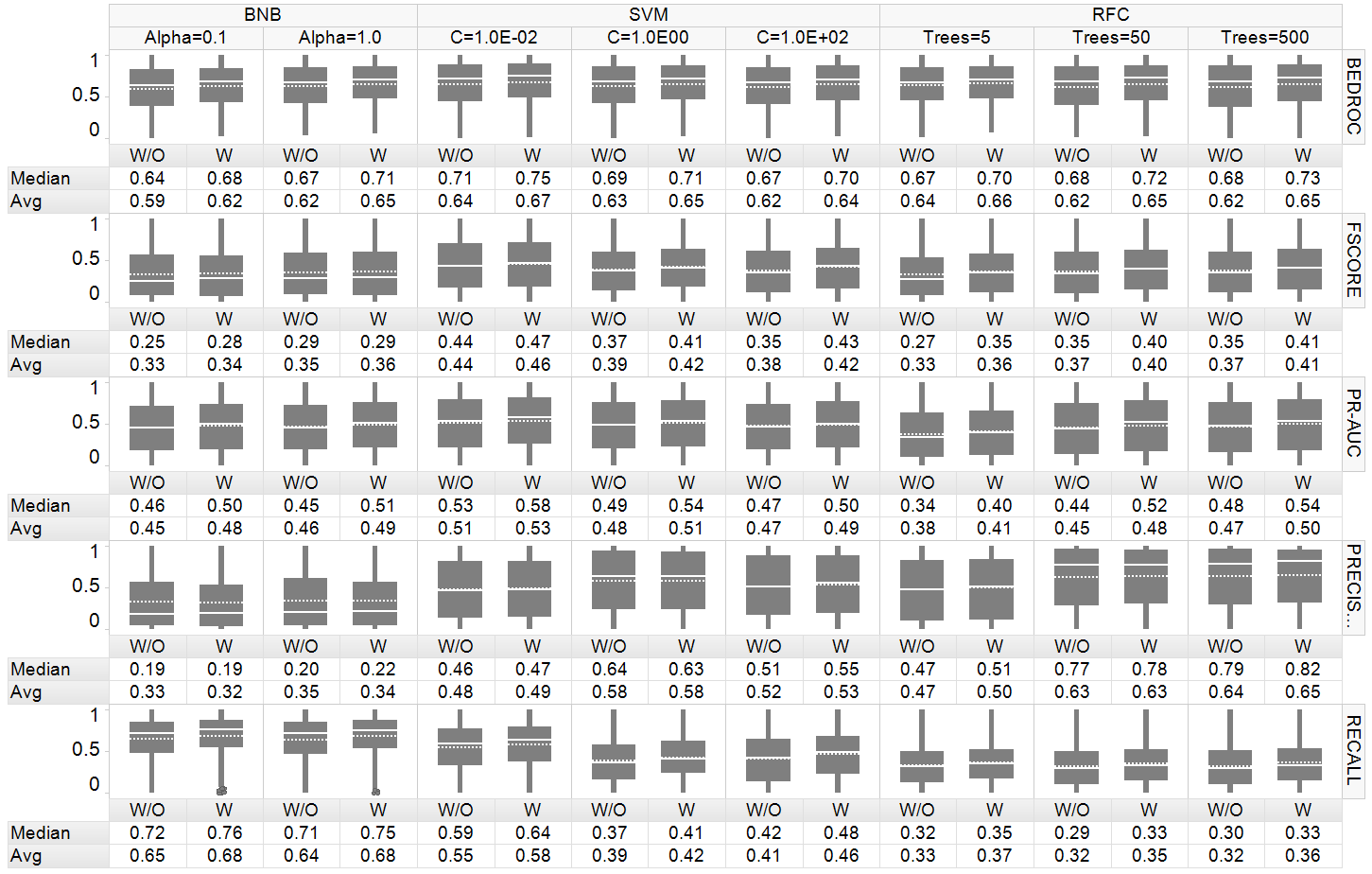

Supplement: Supplementary Figure S8 [file sf8_btx525.png]

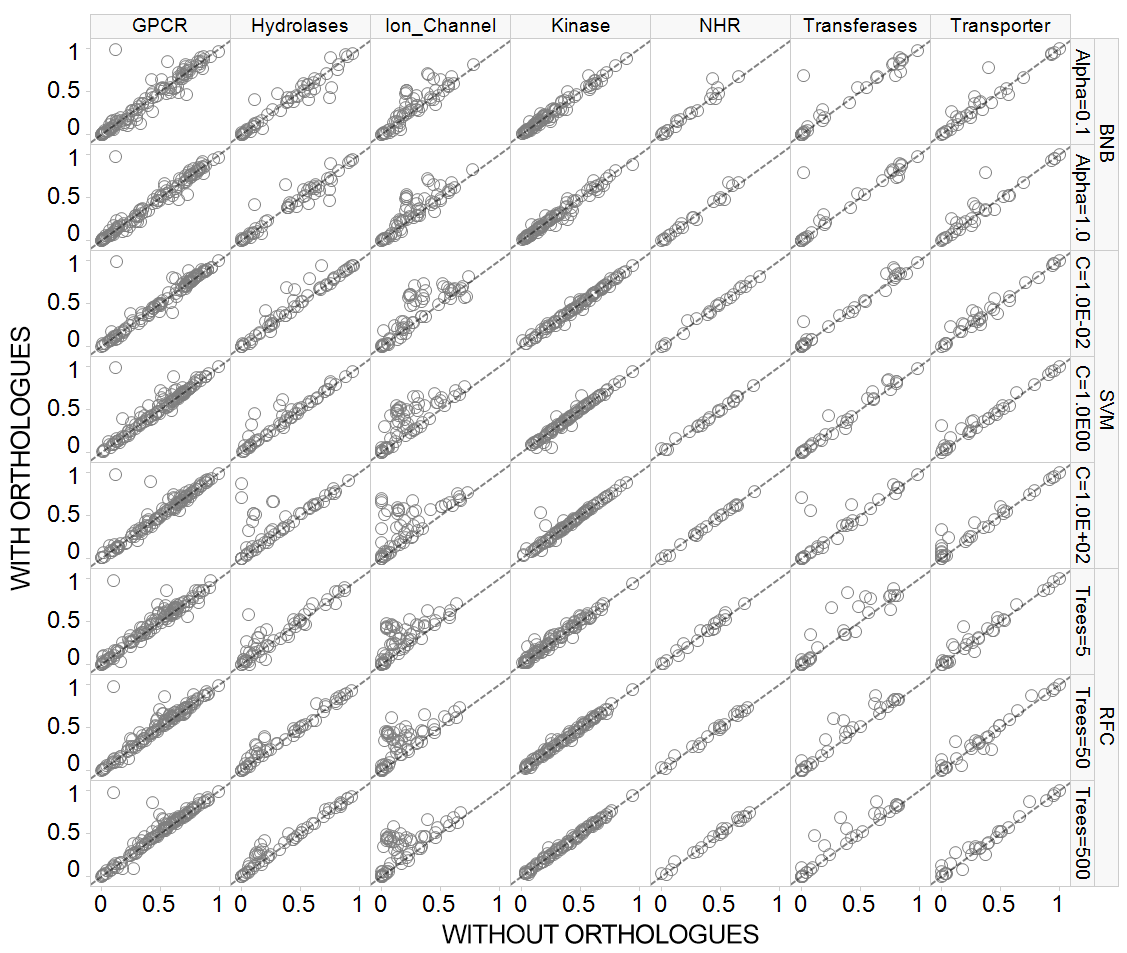

Supplement: Supplementary Figure S9 [file sf9_btx525.png]
