# Supplementary material for: Orthologue chemical space and its influence on target prediction
Source: Bioinformatics. 2017 Aug 26;34(1):72–9. doi: 10.1093/bioinformatics/btx525 (PMC5870859; doi:10.1093/bioinformatics/btx525)
Supplement: Supplementary Table S1 [file st1_btx525.docx]

**Supplementary Material Table S1.** **Orthologue bioactivities table.** The bioactivity data points added when mapping to orthologue targets.

| **ORGANISM** | **GPCR** | **Hydrolases** | **Ion Channel** | **Isomerases** | **Kinase** | **Ligases** | **Lipase** | **Lyases** | **NHR** | **Other** | **Oxidoreductases** | **Phosphatase** | **Protease** | **Transferases** | **Transporter** | **TOTAL** |
| --- | --- | --- | --- | --- | --- | --- | --- | --- | --- | --- | --- | --- | --- | --- | --- | --- |
| *Arabidopsis thaliana* (Mouse-ear cress) | 0 | 1 | 0 | 2 | 0 | 0 | 0 | 0 | 0 | 1 | 15 | 0 | 0 | 0 | 0 | **19** |
| *Bos taurus* (Bovine) | 1563 | 625 | 837 | 0 | 117 | 19 | 202 | 1179 | 48 | 479 | 678 | 5 | 688 | 877 | 55 | **7372** |
| *Canis lupus familiaris* (Dog) (Canis familiaris) | 40 | 118 | 110 | 0 | 1 | 0 | 0 | 0 | 3 | 14 | 146 | 0 | 1 | 0 | 0 | **433** |
| *Danio rerio* (Zebrafish) (Brachydanio rerio) | 0 | 0 | 0 | 0 | 3 | 0 | 0 | 0 | 3 | 1 | 0 | 0 | 0 | 0 | 0 | **7** |
| *Drosophila melanogaster* (Fruit fly) | 6 | 0 | 16 | 1 | 0 | 0 | 0 | 25 | 54 | 4 | 0 | 0 | 0 | 0 | 0 | **106** |
| *Gallus gallus* (Chicken) | 94 | 0 | 4 | 13 | 161 | 0 | 0 | 0 | 5 | 0 | 0 | 0 | 0 | 0 | 4 | **281** |
| *Macaca mulatta* (Rhesus macaque) | 44 | 1 | 0 | 0 | 0 | 0 | 0 | 0 | 0 | 1 | 0 | 0 | 1 | 0 | 40 | **87** |
| *Mus musculus* (Mouse) | 3485 | 430 | 543 | 6 | 1461 | 22 | 242 | 63 | 19666 | 1153 | 837 | 26 | 138 | 612 | 435 | **29119** |
| *Oryza sativa subsp. japonica* (Rice) | 0 | 3 | 0 | 0 | 0 | 0 | 0 | 0 | 0 | 0 | 10 | 0 | 0 | 0 | 0 | **13** |
| *Rattus norvegicus* (Rat) | 30814 | 2419 | 20713 | 253 | 880 | 87 | 750 | 42 | 674 | 1921 | 4009 | 48 | 931 | 1472 | 12143 | **77156** |
| *Saccharomyces cerevisiae* (strain ATCC 204508 / S288c) (Baker's yeast) | 0 | 2 | 0 | 8 | 4 | 1 | 0 | 5 | 0 | 14 | 6 | 0 | 96 | 147 | 2 | **285** |
| *Schizosaccharomyces pombe* (strain 972 / ATCC 24843) (Fission yeast) | 0 | 0 | 0 | 0 | 1 | 0 | 0 | 0 | 0 | 0 | 0 | 0 | 0 | 0 | 0 | **1** |
| **TOTAL** | **36046** | **3599** | **22223** | **283** | **2628** | **129** | **1194** | **1314** | **20453** | **3588** | **5782** | **79** | **1855** | **3108** | **12679** |  |
