# Supplementary material for: Orthologue chemical space and its influence on target prediction
Source: Bioinformatics. 2017 Aug 26;34(1):72–9. doi: 10.1093/bioinformatics/btx525 (PMC5870859; doi:10.1093/bioinformatics/btx525)
Supplement: Supplementary Table S2 [file st2_btx525.docx]

**Supplementary Material Table S2.** **Novel targets added to models**. Inclusion of orthologues enables the modelling of minority classes of targets and alleviates some of the biases in predicted space.

| **Target Classification** | **No. targets available without orthologues** | **No. targets available using orthologues** | **Number of new models added** | **Class Size Increase** |
| --- | --- | --- | --- | --- |
| GPCR | 196 | 201 | 5 | 2.49% |
| Hydrolase | 109 | 120 | 11 | 9.17% |
| Ion Channel | 103 | 111 | 8 | 7.21% |
| Isomerases | 18 | 19 | 1 | 5.26% |
| Kinase | 404 | 405 | 1 | 0.25% |
| Ligase | 15 | 15 | 0 | 0.00% |
| Lipase | 11 | 11 | 0 | 0.00% |
| Lyase | 22 | 22 | 0 | 0.00% |
| NHR | 35 | 35 | 0 | 0.00% |
| Other | 257 | 262 | 5 | 1.91% |
| Oxidoreductase | 93 | 99 | 6 | 6.06% |
| Phosphatase | 28 | 29 | 1 | 3.45% |
| Protease | 137 | 140 | 3 | 2.14% |
| Transferase | 84 | 87 | 3 | 3.45% |
| Transporter | 80 | 87 | 7 | 8.05% |
| **TOTAL** | **1,600** | **1,651** | **51** |  |
