# Supplementary material for: Orthologue chemical space and its influence on target prediction
Source: Bioinformatics. 2017 Aug 26;34(1):72–9. doi: 10.1093/bioinformatics/btx525 (PMC5870859; doi:10.1093/bioinformatics/btx525)
Supplement: Supplementary Table S3 [file st3_btx525.docx]

**Supplementary Material Table S3.** **Pathway and disease coverage.** Orthologue inclusion enables the realized protocol to extend and access new pathway and disease space due to the addition of targets.

| **Name** | **Database** | **Number of Novel Annotations** |
| --- | --- | --- |
| Action Potential Propagation (GO:0098870) | NCBI BioSystems | 2 |
| Macromolecule Depalmitoylation (GO:0098734) | NCBI BioSystems | 2 |
| Neuronal Action Potential Propagation (GO:0019227) | NCBI BioSystems | 2 |
| Protein Depalmitoylation (GO:0002084) | NCBI BioSystems | 2 |
| Amine Transport (GO:0015837) | NCBI BioSystems | 1 |
| Aminergic Neurotransmitter Loading Vesicle (GO:0015842) | NCBI BioSystems | 1 |
| ATP Synthesis Coupled Proton Transport (GO:0015986) | NCBI BioSystems | 1 |
| Cellular Hyperosmotic Response (GO:0071474) | NCBI BioSystems | 1 |
| Endocytic Recycling (GO:0032456) | NCBI BioSystems | 1 |
| Malignant neoplasm of breast | DisGeNET | 22 |
| Breast Carcinoma | DisGeNET | 21 |
| Schizophrenia | DisGeNET | 17 |
| Tobacco Use Disorder | DisGeNET | 17 |
| Carcinogenesis | DisGeNET | 16 |
| Neoplasm Metastasis | DisGeNET | 15 |
| Colorectal Cancer | DisGeNET | 15 |
| Colorectal Carcinoma | DisGeNET | 15 |
| Non-Insulin-Dependent Diabetes Mellitus | DisGeNET | 15 |
| Bipolar Disorder | DisGeNET | 14 |
