# Supplementary material for: Orthologue chemical space and its influence on target prediction
Source: Bioinformatics. 2017 Aug 26;34(1):72–9. doi: 10.1093/bioinformatics/btx525 (PMC5870859; doi:10.1093/bioinformatics/btx525)
Supplement: Supplementary Table S4 [file st4_btx525.docx]

**Supplementary Material Table S4.** **Top conflicting orthologue labels.** The left of the table shows overlap between orthologue actives and human inactive compounds, whilst right shows the number of conflicting bioactivities comparing human actives to orthologue inactives.

|  | **Overlap between Orthologue Actives and Human Inactives** | | | | | | **Overlap between Human Actives and Orthologue Inactives** | | | | | |
| --- | --- | --- | --- | --- | --- | --- | --- | --- | --- | --- | --- | --- |
| **Rank** | Uniprot Mapping | Target Class | CLUSTAL Sequence Similarity % | Orthologue Actives (ChEMBL) | Human Inactives  (PubChem) | No. of Conflicts | Uniprot Mapping | Target  Class | CLUSTAL  Sequence Similarity % | Human Actives  (ChEMBL) | Orthologue Inactives  (PubChem) | No. of Conflicts |
| 1 | P35372/  P33535 | GPCR | 93.75 | 2,291 | 351,686 | 85 | P35610/  O70536 | Transferases | 85.27 | 183 | 290,700 | 138 |
| 2 | P41143/  P33533 | GPCR | 93.56 | 1,603 | 367,570 | 82 | P28223/  P35363 | GPCR | 91.51 | 2,578 | 358,998 | 117 |
| 3 | P11229/  P08482 | GPCR | 98.70 | 1,075 | 357,375 | 75 | P51449/  P51450 | NHR | 88.61 | 422 | 279,378 | 112 |
| 4 | P43681/  P09483 | Ion Channel | 83.07 | 1,384 | 224 | 58 | Q13627/  Q63470 | Kinase | 99.61 | 976 | 307,103 | 88 |
| 5 | P10275/  P15207 | NHR | 84.44 | 324 | 454,011 | 55 | P11229/  P08482 | GPCR | 98.70 | 1,482 | 63,914 | 51 |
| 6 | Q00613/  P38532 | Other | 89.04 | 409 | 407,994 | 45 | Q8NER1/  O35433 | Ion Channel | 85.71 | 1,632 | 282 | 24 |
| 7 | P06401/  Q690N0 | NHR | 93.98 | 46 | 366,682 | 38 | Q07869/  P23204 | NHR | 92.31 | 2,079 | 45 | 20 |
| 8 | Q01959/  P23977 | Transporter | 93.07 | 2,651 | 407,569 | 37 | P21554/  P47746 | GPCR | 97.04 | 3,701 | 59 | 15 |
| 9 | P14416/  P61169 | GPCR | 95.72 | 3,576 | 403,524 | 37 | P14902/  P28776 | Oxido-  reductases | 60.69 | 267 | 45 | 13 |
| 10 | P41143/  P32300 | GPCR | 93.55 | 959 | 367,570 | 36 | O43526/  O88943 | Ion Channel | 92.33 | 136 | 303,929 | 10 |
