# Supplementary material for: Orthologue chemical space and its influence on target prediction
Source: Bioinformatics. 2017 Aug 26;34(1):72–9. doi: 10.1093/bioinformatics/btx525 (PMC5870859; doi:10.1093/bioinformatics/btx525)
Supplement: Supplementary Table S5 [file st5_btx525.docx]

**Supplementary Material Table S5.** **Top discordant pChEMBL values.** Eight of the ten measurements comprise different confidence scores between human and orthologue, suggesting discordance may be exaggerated due to affinity at a protein complex, rather than different affinity at the isolated protein. For example, confidence scores eight and nine denote affinity for the isolated protein, whereas confidence scores five and seven denote pChEMBL affinity at the protein complex.

| **Rank** | **Compound**  **ID** | **Target Class** | **Human Uniprot** | **Ortho Uniprot** | **Orthologue Organism** | **Human pChEMBL** | **Human Confidence Score** | **Ortho pChEMBL** | **Ortho Confidence Score** | **∆pChEMBL** | **Assay**  **Type** | **Activity Unit** |
| --- | --- | --- | --- | --- | --- | --- | --- | --- | --- | --- | --- | --- |
| 1 | CHEMBL  267930 | GPCR | P14416 | P61168 | *Mus musculus* | 10.22 | 8 | 4.46 | 5 | **5.76** | Binding | IC_50_ |
| 2 | CHEMBL  430497 | Other | P36544 | Q05941 | *Rattus norvegicus* | 4.68 | 8 | 10.4 | 5 | **5.72** | Binding | K_i_ |
| 3 | CHEMBL  526688 | Oxido-  reductases | P29475 | P29476 | *Rattus norvegicus* | 9.6 | 8 | 4.36 | 9 | **5.24** | Binding | K_i_ |
| 4 | CHEMBL  59986 | Other | P36544 | Q05941 | *Rattus norvegicus* | 5.05 | 8 | 10.28 | 5 | **5.23** | Binding | K_i_ |
| 5 | CHEMBL  510760 | Oxido-  reductases | P29475 | P29476 | *Rattus norvegicus* | 9.41 | 8 | 4.23 | 9 | **5.18** | Binding | K_i_ |
| 6 | CHEMBL  267930 | GPCR | P21917 | P51436 | *Mus musculus* | 9.48 | 9 | 4.46 | 5 | **5.02** | Binding | IC_50_ |
| 7 | CHEMBL  6623 | Other | P36544 | Q05941 | *Rattus norvegicus* | 5.46 | 7 | 10.39 | 7 | **4.93** | Binding | IC_50_ |
| 8 | CHEMBL  321252 | Transferases | P49354 | P29702 | *Bos Taurus* | 9.92 | 7 | 5 | 7 | **4.92** | Binding | IC_50_ |
| 9 | CHEMBL  327106 | Transferases | P49354 | P29702 | *Bos taurus* | 9.92 | 8 | 5 | 9 | **4.92** | Binding | IC_50_ |
| 10 | CHEMBL  497939 | Other | P36544 | Q05941 | *Rattus norvegicus* | 4.15 | 6 | 9.05 | 9 | **4.9** | Binding | K_i_ |
