# Supplementary material for: Orthologue chemical space and its influence on target prediction
Source: Bioinformatics. 2017 Aug 26;34(1):72–9. doi: 10.1093/bioinformatics/btx525 (PMC5870859; doi:10.1093/bioinformatics/btx525)
Supplement: Supplementary Figure Legends [file sf_index_btx525.docx]

**Supplementary Fig. Index**

**Supplementary Fig. S1: Androgen Receptor superposition.** Human AR (PDB ID: 1T65) (blue), and rat AR (PDB ID: 3G0W) (orange), exhibit similar superposition in the binding site. Conflicting data points between these two orthologous proteins are thus likely due to the misannotation of bioactivities.

**Supplementary Fig. S2: Laboratory pChEMBL concordance.** Human pChEMBL discordance between repeats against same protein within the same organism, before (dark grey) and after (light grey) distinguishing between “binding” and “functional” assay type.

**Supplementary Fig. S3: Species pChEMBL concordance.** Concordance of human and orthologue pChEMBL bioactivities. *Units* within organisms are shown to comprise different linear regression confidence interval profiles, thus one can trust certain measurements varying certain confidence within species. Significant R^2^ scores are annotated with “*”.

**Supplementary Fig. S4: NOS docking study.** Docking studies predicted different molecular interactions for CHEMBL526688 (shown in its most stable protonated form at physiological pH) with the amino acid residues of *Homo Sapiens* Nitric Oxide Synthase (NOS) (PDB ID: 5ADF) and *Rattus Norvegicus* NOS (PDB ID: 5FVO), which provides explanation for difference in activity against each. a) interactions with human NOS: CHEMBL526688 exhibits H-bonds *via* its amino groups with Arg_486_, Glu_597_, Asp_605_, and Arg_608_, the pyridine ring is π-stacked with Arg_486_ and Arg_608_, and the benzene ring is π-stacked with Hem_750_ b) interactions with rat NOS: CHEMBL526688 H-bonds *via* its amino groups with Trp_587_, Glh_592_, and Hem_750_ and the Nitrogen of the pyridine ring with Glh_592_, the pyridine ring is π-stacked with Hem_750_ , and the benzene ring is π-stacked with Arg_596_. Amino acid sequence for the active sites in human and rat NOS was found with MOE.

**Supplementary Fig. S5: Protein similarity and pChEMBL discordance.** The “Stats-prot-change” metric shown on the *x-axis* is used to the define orthologue groups in HomoloGene. The box plot shows the median (block white line) and upper quartile (Q3) and lower interquartile (Q1) ranges by the thick box. The upper and lower adjacent values are shown by the thin lines, whilst outliers are shown via the markers. The histogram distribution is also shown on the vertical axis.

**Supplementary Fig. S6: Distribution of BEDROC, PR-AUC, F_1_-Score, precision and recall scores obtained during time series split validation.** Scores obtained without orthologues are shown in the columns “W/O”, whilst scores obtained with orthologues are shown in the “W” column. The box plot shows the median (block white line), average (dashed white line) and the upper quartile (Q3) and lower interquartile (Q1) ranges by the thick box. The upper and lower adjacent values are shown using thin lines, whilst outliers are shown using circular markers.

**Supplementary Fig. S7: Time series split validation F_1_-Score performance split by target classification.** Scores above the vertical line denote target prediction models which have improved scores due to orthologue inclusion, whilst scores below outline where models have a decrease in performance. The distribution outlines that the effect of orthologue inclusion differs between target classifications, for example kinases show little change in performance, whilst ion channels show large variation in the scores obtained before and after addition of orthologue bioactivity space.

**Supplementary Fig. S8: Distribution of BEDROC, PR-AUC, F_1_-Score, precision and recall scores obtained during AstraZeneca external validation.** The box plot shows the median (block white line), average (dashed white line) and the upper quartile (Q3) and lower interquartile (Q1) ranges by the thick box. The upper and lower adjacent values are shown using thin lines, whilst outliers are shown using circular markers.

**Supplementary Fig. S9: AstraZeneca external validation F1-Score performance split by target classification.** In comparison to cross validation, the external performance shows many models fall above the diagonal line, thus orthologue bioactivity space has been successfully incorporated into the models for these markers.
